# Pharmacokinetics of active compounds of a *Terminalia chebula* Retz. Ethanolic extract after oral administration rats using UPLC-MS/MS

**DOI:** 10.3389/fphar.2023.1067089

**Published:** 2023-01-13

**Authors:** Guangzhe Yao, Xinxin Miao, Mengxuan Wu, Zhenguo Lv, Yu Bai, Yanxu Chang, Huizi Ouyang, Jun He

**Affiliations:** ^1^ Tianjin State Key Laboratory of Component-based Chinese Medicine, Tianjin University of Traditional Chinese Medicine, Tianjin, China; ^2^ Haihe Laboratory of Modern Chinese Medicine, Tianjin, China

**Keywords:** *Terminalia chebula* Retz. extracts, active compounds, pharmacokinetic, UPLC-MS/MS, Rat plasma

## Abstract

*Terminalia chebula* Retz. (TC) is a well-known Chinese herbal medicine and rich in chemical components with multiple pharmacological effects. In this study, an ultra-performance liquid chromatography-tandem mass spectroscopy (UPLC-MS/MS) method was developed and used to determine the blood concentrations of nine active compounds (chebulic acid, gallic acid, protocatechuic acid, corilagin, chebulagic acid, chebulinic acid, 1,2,3,4,6-O-pentagalloylglucose, ellagic acid and ethyl gallate) after oral administration of TC extracts in rats. Pretreatment of plasma samples with protein precipitate with methanol was carried out, and caffeic acid was used as the internal standard (IS). Compounds precisions of intra- and inter-day were less than 14.6%, and the accuracy ranged from −11.7% to 13.5%. The extraction recoveries of compounds were between 84.9% and 108.4%, while matrix effects occurred between 86.4% and 115.9%. Stability tests showed that all nine analytes had been stable under four storage conditions, and statistically significant the relative standard deviations were under 13.7%. The validated UPLC-MS/MS method was applied with great success to plasma pharmacokinetics analysis of the TC extracts, and the pharmacokinetic results showed that among the nine components, the area under the concentration-time curve (AUC_(0-tn)_, 231112.38 ± 64555.20 h ng/mL) and maximum concentration (C_max_, 4,983.57 ± 1721.53 ng/mL) of chebulagic acid were relatively large, which indicated that it had a higher level of plasma exposure. The half-life of elimination (T_1/2_) of chebulinic acid, corilagin and chebulagic acid were 43.30, 26.39 and 19.98 h, respectively, suggesting that these analytes showed prolonged retention and metabolize more slowly *in vivo*. This study would deliver a theoretical foundation for the further application of TC in clinical practice.

## 1 Introduction

*Terminalia chebula* Retz. is to be derived from the well-ripened fruit of the plant in the family Combretaceae, which includes *Terminalia chebula* Retz. or *Terminalia chebula* Retz. var. *tomentella* Kurt (State Pharmacopoeia Commission, 2020). It is always listed at the top of the list of “Ayurvedic Materia Medica” because of its strong detoxifying properties for the body. Moreover, it mainly used for its effects on the gastrointestinal and respiratory systems in Chinese medicine. And it also exists in lots of Tibetan medicine prescription to cure various diseases, which makes its known as the “King of Tibetan Medicine”. With pronounced antibacterial (Kuchma and O’Toole, 2000), neuroprotective (TC alcohol extracts in the concentration range of 6.25–50 μg/ml had no toxic effects on cells, and concentrations above 25 μg/ml significantly increased the viability of quinolinic acid-injured cells.) (Sadeghnia et al., 2017), antioxidant (Kim et al., 2014), anticancer (TC ethyl acetate fraction in the concentration of 50 g/ml alleviated HSC-T6 growth and some anticancer activity.) (Chen et al., 2015), antidiabetic (Kannan et al., 2012), enhancing immunity effect (TC extract (100 mg/kg/p.o.) has better immunomodulatory activity) (Aher et al., 2010) and prevention of atherosclerosis (The concentration of TC at 100 and 250 μg/ml preventede atherosclerosis) (Lee et al., 2015). Because of its rich chemical components, TC can be used to treat sore throat, asthma, vomiting, diarrhea, bleeding and other diseases (Bag et al., 2013; Gaire et al., 2013; Ekambaram et al., 2020).

Modern pharmacological studies showed extensively that tannins such as chebulinic acid, chebulagic acid, ellagic acid, and corilagin, phenolic acids such as gallic acid, protocatechuic acid and ethyl gallate were the main bioactive components underpinning the pharmacological effects of TC (Reddy et al., 1994; Walia and Arora, 2013). For instance, tannins such as chebulinic acid protects the stomach by inhibiting the activity of the H^+^K^+^-ATP (proton pump) enzyme, indicating that it might represent an effective treatment to reduce the incidence of gastric ulcers (Chebulinic acid at a dose of 40 mg/kg has a good anti-gastric ulcer effect) (Mishra et al., 2013). Chebulagic acid inhibits the activation of ERK, JNK, p38, and Akt as well as NF-KB signaling pathways, reduces MDR-1 through COX-2, and has a synergistic effect with azamycin-induced human hepatoma cell toxicity (HepG2) (The concentration of chebulagic acid at 50 μM increased the sensitivity of HepG2 cells towards Dox induced cytotoxicity) (Achari et al., 2011). Furthermore, phenolic acids (gallic acid, protocatechuic acid and ethyl gallate) is an active substance isolated from the TC, which has extensive pharmacological actions such as antioxidant and anti-inflammatory effects (Hazra et al., 2010). It is worth emphasizing that the compounds in TC have far-reaching medicinal value and are worthy of further research and development.

The therapeutic effect of Chinese herbal medicine is based on two key prerequisites, that is, it has sufficient bioavailability and biological effect in the active site after administration, and has the inherent ability to produce the expected effect in its main action forms, namely invariance and metabolism. Because of the chemical complexity of herbal medicine, pharmacokinetics can be used as a screening method to examine whether the components are effectively utilized by the body after administration (Acosta-Estrada et al., 2014; Zhang et al., 2017). Meanwhile, the components that deserve further study would be found, and make a critical step to revealing the material basis of the efficacy of herbal medicine. The bioactive components of TC including chebulic acid, gallic acid, protocatechuic acid, corilagin, chebulagic acid, chebulinic acid, 1,2,3,4,6-O-pentagalloylglucose, ellagic acid and ethyl gallate have good therapeutic effects, such as anti-inflammatory, anti-viral and hypoglycaemic (Li et al., 2018; Shanmuganathan and Angayarkanni, 2018; Song et al., 2020). However, their pharmacokinetics in rats is still to be studied in depth to provide a theoretical basis for the safe clinical use of TC.

Among the compounds isolated and identified from TC, tannins and phenolic acids were found to be a widely studied chemical classes. Meanwhile, the pharmacokinetic profiles of tannins, chebulagic acid and chebulinic acid in rats based on LC-MS/MS methods have been reported (Bei et al., 2013; Lu et al., 2019). However, only the pharmacokinetics and tissue distribution of three monomer components were studied, which could not fully reveal the scientific connotation of TC integrity. Therefore, in this study, a reliable method of UPLC-MS/MS system was established and applied to the pharmacokinetic study of nine bioactive components after oral administration. Through the study of the dynamic changes of multi-compounds *in vivo*, the material basis of TC to exert its efficacy can be fully elucidated, which provided a meaningful reference for the rational usage of TC and the development of safe and effective drugs.

## 2 Materials and method

### 2.1 Chemicals and reagents

Chengdu Desite Bio-Technology Co., Ltd. (Chengdu, China) had a large supply of the standard compounds of chebulic acid, gallic acid, 1,2,3,4,6-O-pentagalloylglucose, protocatechuic acid, corilagin, chebulagic acid, chebulinic acid, ellagic acid, ethyl gallate and caffeic acid (IS) (purity ≥98%). Fisher Scientific (Fair Lawn, NJ, United States) made both methanol and acetonitrile (chromatographic grade) available. Formic acid (chromatographic grade) was provided by ROE (St. Louis, MO, United States). The Millipore-Q water purification system (Tianjin Xinrui Bio-Technology Co., Ltd) was used to produce demineralized water. TC was collected from Anhui province in China.

### 2.2 Chromatographic and mass spectrometry conditions

UPLC-MS/MS system was used for analysis and monitoring, which consisted primarily of an Agilent 1290 UPLC system (Agilent Technologies, Germany) and an Agilent 6,470 series triple quad (TQ) mass spectrometer (MS) (Agilent Technologies, Singapore) equipped with an electrospray ionization source.

Separation was carried out on an ACQUITY UPLC^®^ HSS T3 column (2.1 × 100 mm, 1.8 μm), and temperature in the column was kept at 20°C. In the mobile phase, 0.1% formic acid in water and acetonitrile were used as solvents A and B, respectively. Gradient elution was performed as follows: 1%–5% B at 0–3 min; 5%–30% B at 3–4 min; 30%–40% B at 4–8 min; 40%–100% B at 8–9 min; 100%–100% B at 9–12 min. The flow rate was set at 0.3 ml/min and the injection volume was 10 μL.

Multiple reaction monitoring (MRM) was used for quantitative analysis in negative (−) ionization mode. The mass spectrometric parameters were set as follows: drying gas (N_2_) temperature was 300°C; gas (N_2_) flow rate was 7 L/min; atomizer pressure was 35 psi; sheath gas flow was 11 L/min and capillary voltage was 3800 V. The specific mass spectrometric parameters of the precursor ion, product ion, fragmentor, collision energy, and ion modes of the nine compounds and IS are shown in Table 1.

**TABLE 1:** Mass spectrometry parameters of nine analytes and IS in TC extracts.

Components	Precursor ion (*m/z*)	Product ion (*m/z*)	Fragmentor (V)	Collision energy (V)	Ion mode
Chebulic acid	355.0	337.0	93	12	(−)
Gallic acid	169.0	125.0	113	12	(−)
Protocatechuic acid	153.0	109.0	98	12	(−)
Corilagin	633.1	301.0	161	40	(−)
Chebulagic acid	953.1	301.1	275	44	(−)
Caffeic acid (IS)	179.0	135.0	88	16	(−)
Chebulinic acid	955.1	955.1	219	5	(−)
1,2,3,4,6-O-pentagalloylglucose	939.1	939.1	257	5	(−)
Ellagic acid	301.0	301.0	171	5	(−)
Ethyl gallate	197.0	124.0	123	24	(−)

### 2.3 *Terminalia chebula* Retz. Extracts preparation

Ripe fruits of *Terminalia chebula* Retz are yellowish grey, spherical or ovoid and about one to two inches in size, which has five lines or five ribs on the outer skin. And TC samples of this study were purchased from Bozhou Chinese herbal medicine market in Anhui province and crushed in a grinder and passed through a 60 mesh sieve. The preparation method of TC extracts was as follows: 100.0 g TC was precisely weighed and extracted with ethanol with 30% water under hot reflux for 10 times, each time for an hour. The extracts were then filtered and mixed, which was evaporated and concentrated in the decompressed state, and the TC extracts after drying were finally sprayed into a fine powder, which was stored in desiccant before analysis. Table 2 displayed the content of chebulic acid, gallic acid, protocatechuic acid, corilagin, chebulagic acid, chebulinic acid, 1,2,3,4,6-O-pentagalloylglucose, ellagic acid and ethyl gallate in TC extracts.

**TABLE 2:** The content of nine analytes in TC extracts (*n* = 3).

Components	Content (μg/g)
Chebulic acid	34582.7 ± 1,656.2
Gallic acid	23144.9 ± 183.6
Protocatechuic acid	268.5 ± 28.1
Chebulinic acid	62357.3 ± 1,483.1
Corilagin	13784.9 ± 264.2
Chebulagic acid	47033.7 ± 2,859.2
1,2,3,4,6-O-pentagalloylglucose	2,233.6 ± 231.2
Ellagic acid	14195.2 ± 726.3
Ethyl gallate	481.6 ± 13.3

### 2.4 Preparation of standard solutions, calibration standards and quality control samples

The standard stock solutions of 1 mg/ml were prepared by weighing 10 mg of chebulic acid, gallic acid, protocatechuic acid, corilagin, chebulagic acid, chebulinic acid, 1,2,3,4,6-O-pentagalloylglucose, ellagic acid, ethyl gallate and caffeic acid (IS). Continuous dilution with methanol produced a working solution from the stock solution.

A mixture of 20 μl serial concentration mixed working solutions of standards and 20 μl IS solutions (caffeic acid, 1,000 ng/ml) was added to 100 μl blank plasma to obtain standard curve samples. The final plasma sample concentrations were 100, 250, 500, 1,000, 2000, 5,000, 10000, 20000, 40000 ng/mL for chebulinic acid; 25, 62.5, 125, 250, 500, 1,000, 2,500, 5,000, 10000 ng/ml for chebulic acid, gallic acid, chebulagic acid and corilagin; 1, 2.5, 5, 10, 20, 50, 100, 200, 400 ng/ml for protocatechuic acid, ethyl gallate and 1,2,3,4,6-O-pentagalloylglucose; and 10, 25, 50, 100, 200, 500, 1,000, 2000, 4,000 ng/ml for ellagic acid.

Preparation of quality control (QC) samples with three concentrations (low, medium, and high) of 16, 160, and 3,200 ng/ml for ellagic acid; 1.6, 16, 320 ng/ml for protocatechuic acid, ethyl gallate and 1,2,3,4,6-O-pentagalloylglucose; 40, 400 and 8,000 ng/ml for chebulic acid, gallic acid, corilagin and chebulinic acid and 160, 1,600, 32000 ng/ml for chebulagic acid. Both QC samples and stock solutions were stored in a refrigerator at 4°C.

### 2.5 Plasma sample preparation

The plasma sample (100 μl) was sequentially spiked with 20 μl IS solutions (caffeic acid, 1,000 ng/ml); 20 μL methanol, and 10 μl formic acid, then vortex-mixed for 1 min. The mixture was precipitated protein with 1,000 μl methanol by vortex mixing for 3 min. The supernatant was collected into a 1.5 ml clean centrifuge tube after centrifugation at for 10 min 14,000 rpm, and evaporated under a nitrogen stream until dry. After that, the residue was redissolved in 100 μl methanol, vortex-mixed for 3 min, then centrifuged for 10 min at 14,000 rpm. 10 μl analyte was injected into the UPLC-MS/MS system.

### 2.6 Method validation

#### 2.6.1 Specificity

The specificity was assessed by way of paralleling chromatograms of blank plasma samples, corresponding blank plasma samples spiked with analytes and IS, as well as plasma samples from the rats collected after oral administration of TC extracts.

#### 2.6.2 Linearity and lower limit of quantification

A calibration curve was derived by plotting each analyte’s peak area ratio to IS *versus* its concentration. The regression relationship is described by linear regression equation with weighting coefficient of 1/*X*. The signal-to-noise ratio (S/N) is defined as approximately 10 based on the lower limits of quantification (LLOQ) of the baseline noise assessment.

#### 2.6.3 Precision and accuracy

Six replications of QC samples at three concentrations (low, medium, and high) were analyzed within 1 day or over three consecutive days to assess precision and accuracy. The accuracy was assessed by calculating the relative error (RE %), while intra and inter-day precision were defined as the relative standard deviation (RSD).

#### 2.6.4 Extraction recovery and matrix effect

The extraction recoveries and matrix effects were all investigated by six replicates at the three concentrations mentioned above low, medium and high. The recovery was then determined by comparison of the peak areas of the analytes in the pre-precipitated and post-precipitated samples of the spiked standards. While matrix effects were evaluated by calculating the area of the peak of the analyte in the precipitated spiked sample compared to the area of the peak in the standard solutions.

#### 2.6.5 Stability

Under variable treatment conditions: stored for 12 h at auto-sampler, for 4 hours at room temperature, under three freeze-thaw cycles, and kept at −80°C for 7 days, quality control samples were analyzed to assess the stability of the nine analytes in plasma.

### 2.7 Pharmacokinetic study

Male Sprague-Dawley rats (220 ± 10) g from the Beijing HUAFUKANG Bioscience Co., Inc. (Beijing, China) were selected. In this study, six of male rats were used, given free access to water, and fasted for 12 h before the experimental period. The TC extracts were dissolved in 0.5% CMC-Na aqueous solution as a suspension at a concentration of 1 g/ml. A dose of 10 g/kg of the suspension was administered to the rats orally, the blood samples of approximately 300 μL were drawn from the fundus venous plexus of rats pre- and post-oral administration at 0.00, 0.08, 0.17, 0.25, 0.50, 0.75, 1, 2, 4, 6, 8, 10, 12, 24, 36, 48, 60, 72, 84, 96, 120, 144 and 168 h, respectively. For analysis, the plasma supernatants collected from the centrifuged plasma were frozen at −80°C after centrifugation for 10 min at 6,000 rpm.

### 2.8 Data analysis

Data were expressed as mean ± standard deviation (mean ± SD). MassHunter Workstation software (version B.09.00) was quantitatively used to calculate the plasma concentrations of nine analytes. To evaluate the specific pharmacokinetic parameters, DAS Software (DAS 3.0, Medical College of Wannan, China) was used to process the pharmacokinetic results.

## 3 Results and discussions

### 3.1 Optimization of chromatographic and mass spectrometry conditions

**FIGURE 1:**
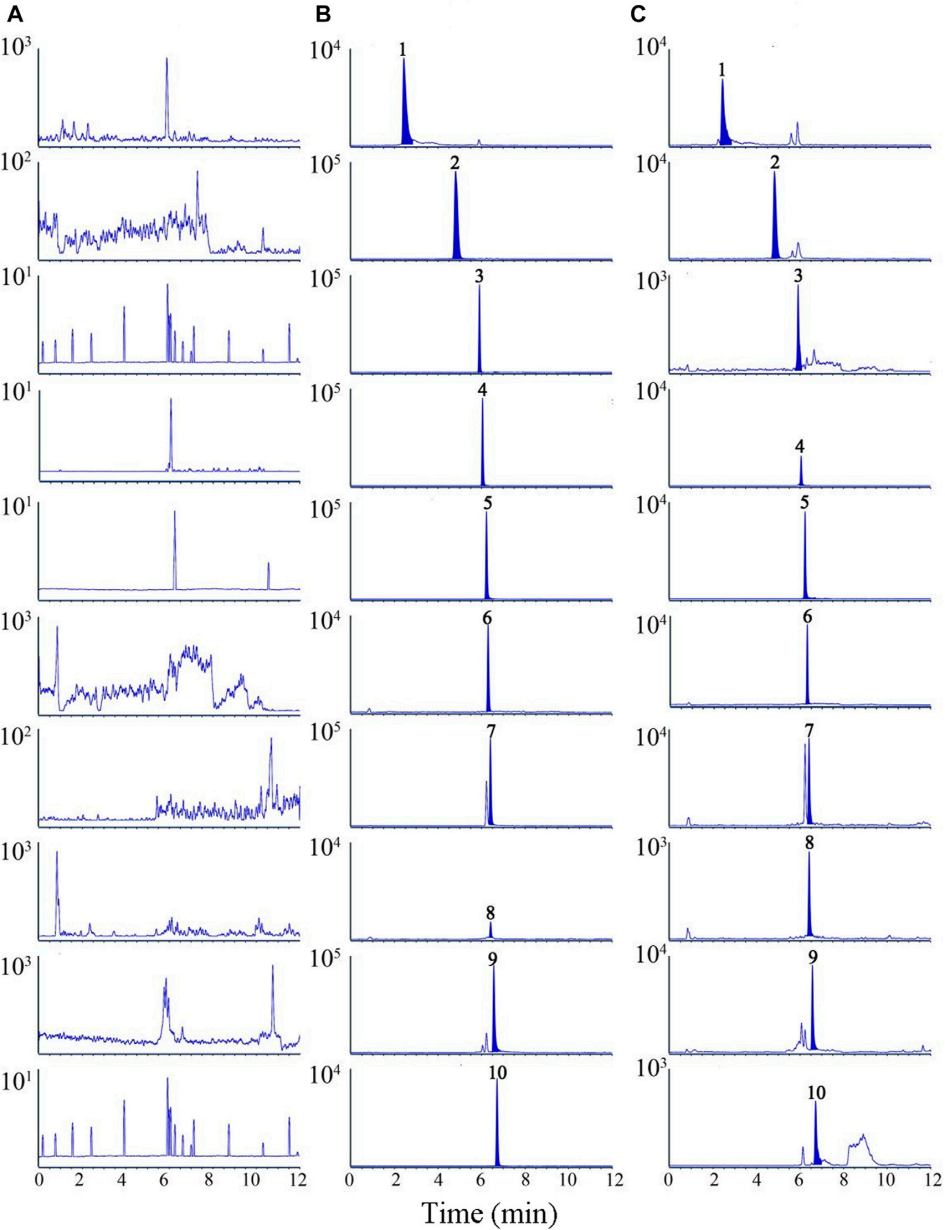
Representative multiple reaction monitoring (MRM) chromatograms of nine analytes and IS in rat plasma samples. **(A)** blank plasma sample, **(B)** blank plasma spiked with nine analytes and IS, **(C)** plasma samples after oral administration of TC extracts. 1. chebulic acid, 2. gallic acid, 3. protocatechuic acid, 4. corilagin, 5. chebulagic acid, 6. caffeic acid (IS), 7. chebulinic acid, 8.1,2,3,4,6-O-pentagalloylglucose, 9. ellagic acid, 10. ethyl gallate.

Suitable chromatographic conditions are important for the analysis of nine compounds. The effects of ACQUITY UPLC^®^ BEH C 18 column (2.1 × 100 mm, 1.7 μm) and ACQUITY UPLC^®^ HSS T3 column (2.1 × 100 mm, 1.8 μm) on chromatographic peaks and elution time were studied experimentally. Meanwhile, water (0.1% formic acid)-acetonitrile and water (0.1% formic acid)-methanol were compared to determine the optimum mobile phase. The results displayed that water (0.1% formic acid)-acetonitrile flow has a better peak shape and lower background noise than water (0.1% formic acid)-methanol flow. Nine analytes and IS were purged in 12 min using ACQUITY UPLC^®^ HSS T3 column at a flow phase flow rate of 0.3 mL/min, as shown in Figure 1. No interference peaks were observed.

A comparison of electrospray ionisation sources in positive and negative ionisation modes was carried out. The results showed the target substances in negative ion mode are more responsive and stable. MS/MS ion conversion monitoring in multiple reaction monitoring (MRM) mode improves the specificity of the detection method. Therefore, the nine analytes were quantitated with electron spray ionization (ESI) in the negative ionisation mode.

### 3.2 Sample preparation

Protein precipitation with methanol acetonitrile and liquid-liquid extraction with ethyl acetate were used to find a suitable method for preparing plasma samples. The results showed that the methanol precipitation protein method had good extraction recoveries for the nine analytes and the matrix effects met the assay requirements. Thus, the protein precipitation method using methanol was used in the sample preparation. Furthermore, the effects of different volumes (400 μl, 600 μl, 800 μl, 1,000 μl) of precipitation solvent on extraction recovery and matrix effect were investigated, and it was found that when the volume of methanol was 1,000 μl, the extraction recovery was between 84.9% and 108.4%, and the matrix effect was between 86.4% and 115.9%, indicating that protein precipitation with methanol could obtain satisfactory extraction recovery and matrix effect.

### 3.3 Method validation

#### 3.3.1 Specificity

**FIGURE 2:**
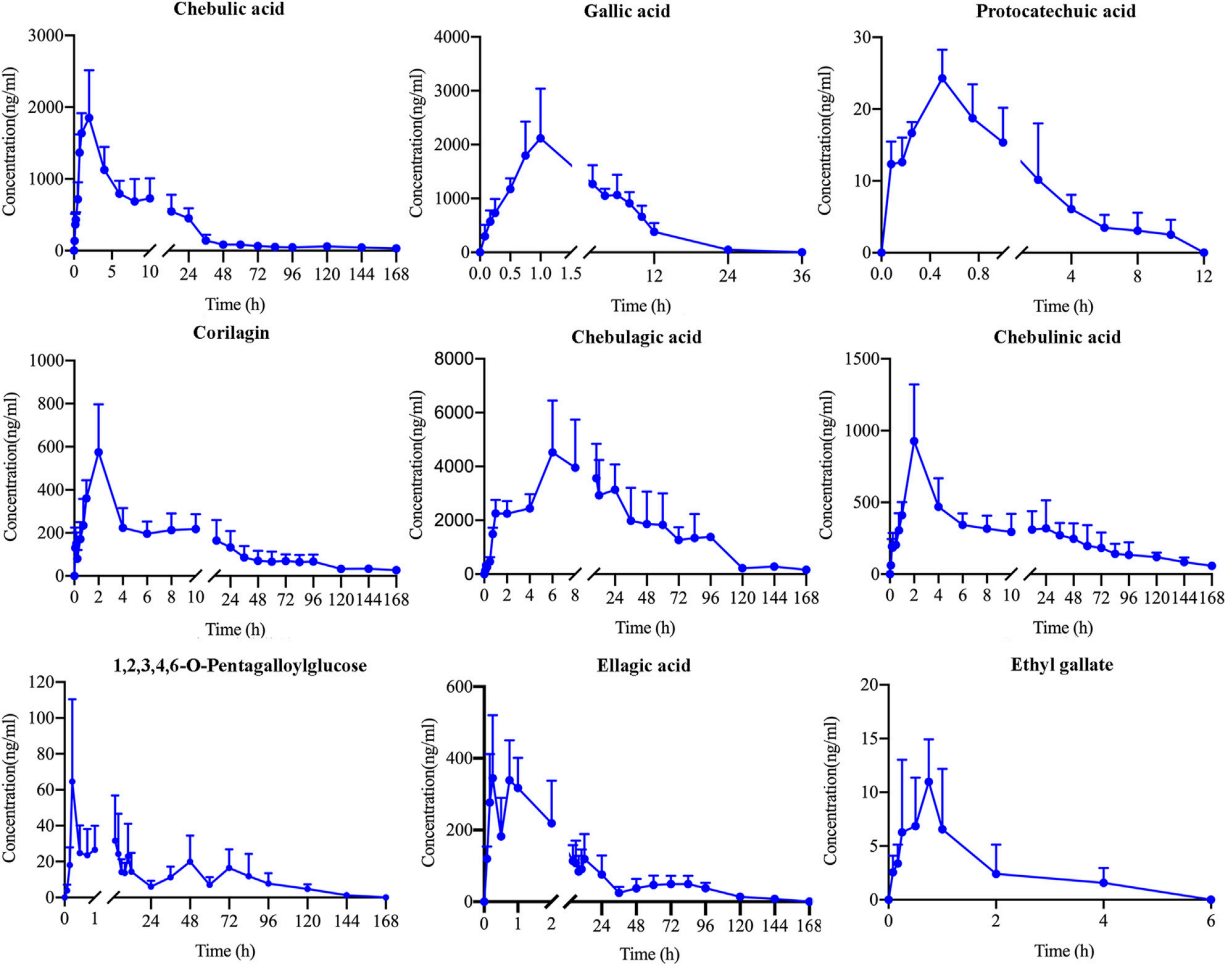
Mean plasma concentration-time profiles of chebulic acid, gallic acid, protocatechuic acid, corilagin, chebulagic acid, chebulinic acid, 1,2,3,4,6-O-pentagalloylglucose, ellagic acid and ethyl gallate in rats after oral administration of TC extracts.

Figure 2 is an MRM chromatogram of a blank plasma sample (A), a blank plasma sample with the addition of nine analytes and IS (B), and a plasma sample taken after oral administration of TC extract (C). As against the sample without reference solution, the samples of standard solution with a certain concentration and the medicated plasma were no obvious interference peaks in the residence time of all the analyzed substances.

#### 3.3.2 Linearity and lower limits of quantification

Table 3 displayed the regression equations, linear ranges, correlation coefficients and LLOQs for the nine analytes. The results indicate that the calibration curves for these analytes showed good linearity (*r* ^2^ > 0.991) over the corresponding concentration ranges. The LLOQs of chebulic acid, chebulinic acid, protocatechuic acid, corilagin, chebulagic acid, gallic acid, 1,2,3,4,6-O-pentagalloylglucose, ellagic acid, ethyl gallate were 12.0, 17.0, 0.4, 0.1, 8.0, 0.9, 0.1, 8.5 and 0.2 ng/mL, separately.

**TABLE 3:** Calibration profiles, correlation coefficients, linear ranges and LLOQs of the nine analytes.

Components	Calibration curve	Correlation coefficients (*r* ^2^)	Linear range (ng/mL)	LLOQs (ng/mL)
Chebulic acid	Y = 0.1033 X - 0.0142	0.991	25–10000	12.0
Gallic acid	Y = 0.4475 X - 0.0117	0.998	25–10000	17.0
Protocatechuic acid	Y = 2.2846 X + 0.0304	0.997	1–400	0.4
Chebulinic acid	Y = 0.0329 X - 0.0057	0.995	25–10000	0.9
Corilagin	Y = 0.221 X - 0.0155	0.998	25–10000	0.1
Chebulagic acid	Y = 0.1816 X + 0.0078	0.996	100–40000	8.0
1,2,3,4,6-O-pentagalloylglucose	Y = 0.0982 X + 0.0015	0.994	1–400	0.1
Ellagic acid	Y = 1.764 X + 0.1248	0.997	10–4,000	8.5
Ethyl gallate	Y = 1.4336 X - 0.0014	0.999	1–400	0.2

#### 3.3.3 Precision and accuracy

Table 4 showed intra-day, inter-day and accurate results of QC samples with low, medium and high concentrations in rat biological matrix. It was less than 11.6% for the RSD of intraday, and it was between −9.3% and 13.3% for the RE of intraday. The daily RSD was less than 14.6%, and the daily RE was between −11.7% and 13.5%. Based on the results, this method has an acceptable level of precision and accuracy.

**TABLE 4:** Precision and accuracy of nine analytes in rat plasma (*n* = 6).

Components	Spiked concentration (ng/mL)	Intra-day			Inter-day		
		Measured (ng/mL)	RE (%)	RSD (%)	Measured (ng/mL)	RE (%)	RSD (%)
Chebulic acid	40	41.7 ± 1.5	4.3	3.6	38.8 ± 3.5	−3.0	8.9
	400	424.3 ± 23.4	6.1	5.5	414.4 ± 51.3	3.6	12.4
	8,000	8,808.9 ± 553.0	10.1	6.3	8,188.2 ± 435.7	2.4	5.3
Gallic acid	40	36.9 ± 4.3	−7.7	11.6	38.1 ± 4.6	−4.7	12.2
	400	430.0 ± 12.9	7.5	3.0	432.2 ± 14.2	8.0	3.3
	8,000	8,507.6 ± 466.2	6.3	5.5	8,288.1 ± 610.2	3.6	7.4
Chebulinic acid	40	43.9 ± 4.8	9.8	11.0	36.0 ± 3.3	−10.1	9.1
	400	366.9 ± 25.1	−8.3	6.8	379.7 ± 12.4	−5.1	3.3
	8,000	7,283.4 ± 194.3	−9.0	2.7	9,079.5 ± 538.1	13.5	5.9
Corilagin	40	40.1 ± 2.8	0.2	6.9	37.4 ± 3.4	−6.5	9.1
	400	433.9 ± 36	8.5	8.3	386.1 ± 56.3	−3.5	14.6
	8,000	7,897.8 ± 504.5	−1.3	6.4	8,457.2 ± 599.9	5.7	7.1
Chebulagic acid	160	162.0 ± 17.9	1.2	11.1	163.9 ± 14.4	2.5	8.8
	1,600	1,450.8 ± 72.5	−9.3	5.0	1,413.4 ± 161.5	−11.7	11.4
	32000	30860.5 ± 2,185.4	−3.6	7.1	36016.4 ± 2006.2	12.6	5.6
Protocatechuic acid	1.6	1.8 ± 0.1	11.5	8.2	1.7 ± 0.2	6.8	10.1
	16	15.2 ± 1.4	−4.8	9.0	15.9 ± 2.1	−0.6	13.2
	320	306.3 ± 15.5	−4.3	5.1	347.6 ± 16.6	8.6	4.8
1,2,3,4,6-O-pentagalloylglucose	1.6	1.6 ± 0.2	1.3	10.1	1.6 ± 0.1	−0.7	8.0
	16	15.9 ± 1.7	−0.6	10.6	16.2 ± 0.8	1.5	5.1
	320	329.6 ± 26.3	3.0	8.0	351.2 ± 28.3	9.8	8.1
Ellagic acid	16	18.1 ± 0.8	13.3	4.3	15.7 ± 2.0	−2.0	13.0
	160	174.1 ± 15.2	8.8	8.8	155.5 ± 9.5	−2.8	6.1
	3,200	3,543.1 ± 204.5	10.8	5.8	3,446 ± 277.4	7.7	8.0
Ethyl gallate	1.6	1.7 ± 0.2	7.6	9.9	1.5 ± 0.2	−6.2	11.1
	16	15.8 ± 1.5	−1.3	9.6	17.9 ± 1.5	11.9	8.5
	320	336.9 ± 30.5	5.3	9.0	330.5 ± 18.5	3.3	5.6

#### 3.3.4 Extraction recovery and matrix effect

Table 5 summarizes the recoveries of analytes at low, medium and high concentrations and their matrix effects. As a result, total analytes had extraction recoveries ranging from 84.9% to 108.4% (RSD <13.5%). Matrix effects varied from 86.4% to 115.9% (RSD <14.6%). These results showed the accuracy and acceptability of extraction recovery and matrix effects.

**TABLE 5:** Extraction recovery and matrix effects of nine analytes in rat plasma (n = 6).

Components	Spiked concentration (ng/mL)	Extraction recovery (%)	RSD (%)	Matrix effect (%)	RSD (%)
Chebulic acid	40	95.7 ± 13.5	13.5	102.4 ± 9.2	9.0
	400	88.0 ± 5.3	5.3	103.6 ± 8.1	7.8
	8,000	96.0 ± 10.7	10.7	109.6 ± 14.1	13.8
Gallic acid	40	92.1 ± 10.8	11.7	106.0 ± 7.7	7.3
	400	100.5 ± 8.3	8.3	97.1 ± 7.9	8.2
	8,000	97.4 ± 10.5	10.8	107.7 ± 9.9	9.2
Chebulinic acid	40	108.4 ± 12.3	11.3	99.7 ± 11.0	11.0
	400	100.1 ± 6.5	6.5	86.4 ± 3.1	3.6
	8,000	101.8 ± 11.9	11.7	104.4 ± 6.1	5.9
Corilagin	40	92.0 ± 9.9	10.8	114.4 ± 11.0	9.6
	400	104.8 ± 8.4	8.0	99.6 ± 1.4	1.4
	8,000	93.3 ± 7.8	8.3	93.7 ± 8.9	9.5
Chebulagic acid	160	101.1 ± 12.4	12.2	104.6 ± 9.6	9.2
	1,600	95.7 ± 9.9	10.3	94.4 ± 6.7	7.1
	32000	99.9 ± 1.9	1.9	88.4 ± 6	6.8
Protocatechuic acid	1.6	84.9 ± 4.4	5.2	111.2 ± 5.8	5.2
	16	92.8 ± 10.3	11.1	115.9 ± 4.6	4.0
	320	94.2 ± 10.1	10.7	99.2 ± 4.6	4.7
1,2,3,4,6-O-pentagalloylglucose	1.6	101.1 ± 10.2	10.1	95.3 ± 8.1	8.5
	16	101.6 ± 9.4	9.2	91.3 ± 11.1	12.1
	320	94.3 ± 11.8	12.5	90.4 ± 6.9	7.6
Ellagic acid	16	95.1 ± 5	5.2	93.0 ± 4.0	4.3
	160	88.7 ± 2.7	3.1	104.2 ± 8.1	7.7
	3,200	90.7 ± 8.2	9.1	98.6 ± 7.1	7.2
Ethyl gallate	1.6	99.8 ± 5.3	5.3	90.5 ± 13.2	14.6
	16	98.9 ± 10.7	10.8	88.9 ± 8.3	9.4
	320	104.6 ± 8.5	8.2	107.9 ± 10.9	10.1

#### 3.3.5 Stability

Table 6 gave the stability evaluation results of QC samples under different conditions. The sample was stabilized at room temperature for 4 h, in an automatic sampler for 12 h and at −80°C for 7 days. The results showed that nine analytic substances in rat plasma can be determined with UPLC-MS/MS, and the relative standard deviation is less than 13.7%.

**TABLE 6:** Stability of nine analytes in rat plasma (*n* = 6).

Components	Spiked concentration (ng/mL)	Room temperature for 4 h		Autosampler for 12 h		Three freeze-thaw cycles			−80°C for 7 days	
		Measured (ng/mL)	RSD (%)	Measured (ng/mL)	RSD (%)	Measured (ng/mL)	RSD (%)		Measured (ng/mL)	RSD (%)
Chebulic acid	40	39.9 ± 1.9	4.6	35.6 ± 3.9	12.2	40.1 ± 3.3	8.3	37.5 ± 2.1		5.6
	400	395.7 ± 29.7	7.5	393.3 ± 52.6	13.4	398.4 ± 12.2	3.1	364.4 ± 17.5		4.8
	8,000	7,996.4 ± 517.7	6.5	8,230.9 ± 726.4	9.3	7,691 ± 316.7	4.1	6,878.6 ± 535.3		7.8
Gallic acid	40	35.5 ± 1.0	2.9	34.9 ± 5.1	12.5	41.0 ± 2.8	6.9	41.2 ± 0.8		2.1
	400	385 ± 36.1	9.4	431.7 ± 27.3	7.2	393.9 ± 35.3	9.0	358.6 ± 21.8		6.1
	8,000	8,051.2 ± 472.7	5.9	8,051.6 ± 602.3	7.7	8,222.3 ± 456.3	5.5	8,304.7 ± 418.5		5.0
Protocatechuic acid	1.6	1.6 ± 0.1	6.3	1.7 ± 0.2	12.5	1.8 ± 0.1	5.9	1.5 ± 0.2		11.7
	16	16.4 ± 0.6	4.0	14.5 ± 2.0	13.6	13.9 ± 0.7	5.0	13.1 ± 1.2		9.1
	320	297.9 ± 8.4	2.8	305.9 ± 15.9	5.3	330.3 ± 18.5	5.6	334.2 ± 8.0		2.4
Corilagin	40	39.6 ± 3.0	7.6	38.8 ± 1.2	3.1	42.8 ± 2.4	5.6	39.7 ± 2.1		5.3
	400	398.2 ± 34.3	8.6	435.7 ± 34.5	8.0	405.3 ± 6.0	1.5	395.6 ± 7.2		1.8
	8,000	8,930.5 ± 535.3	6.0	7,335.4 ± 704.5	8.7	8,802.7 ± 713.0	8.1	8,304.6 ± 362.4		4.4
Chebulagic acid	160	160.8 ± 6.2	3.8	145.5 ± 16.4	12.7	167.9 ± 7.8	4.6	168.0 ± 4.2		2.5
	1,600	1,465.3 ± 158.2	10.8	1,448.9 ± 70.5	4.7	1,391.9 ± 61.5	4.4	1,512.8 ± 65.1		4.3
	32000	31970.0 ± 1,256.3	3.9	34277.3 ± 2,721.2	7.2	33770.8 ± 3,365.5	10.0	31615.2 ± 953.8		3.0
Chebulinic acid	40	36.8 ± 3.9	10.7	42.2 ± 5.1	13.7	41.8 ± 2.3	5.5	47.1 ± 1.6		3.5
	400	414.3 ± 21.9	5.3	359.7 ± 18.1	5.2	378.1 ± 29.2	7.7	373.5 ± 23.6		6.3
Chebulinic acid	8,000	8,592.3 ± 543.8	6.3	7,174.2 ± 321.2	4.5	8,447.4 ± 574.6	6.8	8,787.4 ± 801		9.1
1,2,3,4,6-O-pentagalloylglucose	1.6	1.8 ± 0.1	4.5	1.7 ± 0.2	12.2	1.7 ± 0.2	9.6	1.7 ± 0.2		12.8
	16	16.1 ± 1.0	6.1	17.9 ± 1.4	7.5	17.1 ± 0.9	5.0	16.1 ± 1.7		10.9
	320	341.5 ± 17.1	5.0	349.5 ± 30.6	7.9	336.0 ± 15.9	4.7	333.1 ± 18.4		5.5
Ellagic acid	16	17.5 ± 1.7	9.9	17.6 ± 1.6	11.0	16.9 ± 1.4	8.1	16.8 ± 1.0		5.7
	160	156.3 ± 17.8	11.4	154.8 ± 13.9	9.3	171.8 ± 10.5	6.1	163.8 ± 4.3		2.6
	3,200	3,437.2 ± 188	5.5	3,450.0 ± 313.1	9.2	3,438.7 ± 285.7	8.3	3,594.0 ± 81.5		2.3
Ethyl gallate	1.6	1.4 ± 0.1	6.6	1.7 ± 0.2	8.5	1.6 ± 0.1	6.9	1.6 ± 0.1		7.1
	16	14.6 ± 1.9	13.0	16.3 ± 2.6	13.4	13.6 ± 1.2	9.1	14.2 ± 0.4		3.0
	320	345.6 ± 14.7	4.3	323.3 ± 13.8	4.2	328.6 ± 17.1	5.2	307.4 ± 29.5		9.6

### 3.4 Pharmacokinetic study

This study examined the pharmacokinetics of nine components of TC extract administered intragastrically to rats. Figure 2 depicted the blank plasma concentration-time curve and Table 7 summarizes the corresponding pharmacokinetic parameters.

**TABLE 7:** Pharmacokinetic parameters of nine analytes in normal administration and model administration groups of rats. (Mean ± SD, *n* = 6).

Components	T_max_ (h)	C_max_ (ng/mL)	T_1/2_ (h)	MRT _(0-tn)_ (h)	MRT _(0-∞)_ (h)	AUC _(0-tn)_ (h·ng/mL)	AUC _(0-∞)_ (h·ng/mL)
Chebulic acid	1.67 ± 0.52	1927.06 ± 572.23	6.60 ± 4.36	33.56 ± 3.77	77.92 ± 22.14	29045.30 ± 4,395.44	34069.03 ± 6,641.84
Gallic acid	1.17 ± 0.41	2,234.40 ± 1,201.26	1.51 ± 0.43	6.58 ± 1.07	6.58 ± 1.07	15996.49 ± 5,401.08	15996.75 ± 5,400.92
Protocatechuic acid	0.54 ± 0.10	24.67 ± 3.27	2.06 ± 0.76	3.39 ± 0.83	3.39 ± 0.83	70.82 ± 16.64	70.82 ± 16.64
Corilagin	1.67 ± 0.52	591.06 ± 202.52	26.39 ± 22.25	53.45 ± 4.12	99.39 ± 46.24	16682.95 ± 12405.92	17436.04 ± 12513.04
Chebulagic acid	5.67 ± 3.67	4,983.57 ± 1721.53	19.98 ± 2.51	49.78 ± 4.87	51.84 ± 5.30	231112.38 ± 64555.20	234456.50 ± 65717.01
Chebulinic acid	1.83 ± 0.41	852.55 ± 433.35	43.30 ± 13.20	58.23 ± 5.63	75.99 ± 15.34	38222.29 ± 16301.82	41899.29 ± 15786.18
1,2,3,4,6-O-pentagalloylglucose	0.33 ± 0.20	68.00 ± 41.12	6.52 ± 4.39	55.97 ± 3.73	56.14 ± 3.81	1,489.35 ± 578.46	1,490.93 ± 577.72
Ellagic acid	0.97 ± 0.84	390.28 ± 80.48	8.23 ± 8.95	45.85 ± 8.13	56.55 ± 12.99	6,814.05 ± 1,499.06	7,338.94 ± 1,576.66
Ethyl gallate	0.63 ± 0.26	12.49 ± 4.38	2.08 ± 2.16	1.55 ± 0.420	1.60 ± 0.40	16.86 ± 8.28	16.93 ± 8.20

The T_1/2_ values of 1,2,3,4,6-O-pentagalloylglucose, chebulic acid, gallic acid, protocatechuic acid, ellagic acid and ethyl gallate were 6.60, 1.51, 2.06, 6.52, 8.23 and 2.08 h, separately, these analytes were shown to be eliminated shortly after oral administration. However, the T_1/2_ values of corilagin, chebulagic acid and chebulinic acid were 26.39, 19.98, 43.30 h, separately, and their mean residence time (MRT) were 53.45 ± 4.12, 49.78 ± 4.87 and 58.23 ± 5.63 h, separately, which demonstrated that these three analytical substances existed inside the body for a comparatively long term and may have a continuous treatment effect and enhance the clinical efficacy (Ma, 2015; Gao et al., 2016). The AUC_(0-tn)_ and AUC _(0-∞)_ values of chebulic acid, gallic acid, corilagin, chebulagic acid and chebulinic acid were a lot higher than the others, suggesting that these analytes had higher levels of plasma exposure. And the C_max_ of chebulic acid, gallic acid and chebulinic acid were 1927.06 ± 572.23, 2,234.40 ± 1,201.26 and 852.55 ± 433.35 ng/ml, respectively, suggesting that the concentration of those compounds was high. In combination with AUC results, chebulic acid, gallic acid and chebulinic acid might be the potential active compounds leading to the effect of TC *in vivo*.

Furthermore, it was found that ellagic acid and 1,2,3,4,6-O-pentagalloylglucose had double peaks phenomenon on the blank plasma concentration-time curves in Figure 2. This phenomenon may be caused by hepatointestinal circulation. And, perhaps because of the complexity of the gastrointestinal environment, where are multiple sites of absorption at different points, and their permeability of the ingredients is different (Metsugi et al., 2007; Shin et al., 2009; Müller, and Kanfer, 2011).

## 4 Conclusion

In this study, a simple UPLC-MS/MS method was established and applied to study the pharmacokinetics of 10 g/kg TC extract in rats after a single oral administration. The method was verified, and the detection ability of a low concentration for nine compounds was demonstrated. (chebulic acid, gallic acid, corilagin, protocatechuic acid, chebulagic acid, chebulinic acid, ellagic acid, ethyl gallate and 1,2,3,4,6-O-pentagalloylglucose) with protein precipitation method. The results indicated that the blood concentration and plasma exposure of chebulic acid, gallic acid, corilagin, chebulagic acid and chebulinic acid were higher than other compounds. In addition, compared to the other six components, corilagin, chebulagic acid and chebulinic acid showed prolonged retention and metabolize more slowly. The data obtained in this study can provide useful information for preclinical application, and also provide theoretical basis for the further development of TC.

## Data Availability

The original contributions presented in the study are included in the article/supplementary material, further inquiries can be directed to the corresponding author.
